# A Bayesian analysis of heart rate variability changes over acute episodes of bipolar disorder

**DOI:** 10.1038/s44184-024-00090-x

**Published:** 2024-10-03

**Authors:** Filippo Corponi, Bryan M. Li, Gerard Anmella, Clàudia Valenzuela-Pascual, Isabella Pacchiarotti, Marc Valentí, Iria Grande, Antonio Benabarre, Marina Garriga, Eduard Vieta, Stephen M. Lawrie, Heather C. Whalley, Diego Hidalgo-Mazzei, Antonio Vergari

**Affiliations:** 1 School of Informatics, University of Edinburgh, Edinburgh, UK; 2 Bipolar and Depressive Disorders Unit, Hospìtal Clinic de Barcelona, Barcelona, Spain; 3 Institut d’Investigacions Biomèdiques August Pi i Sunyer (IDIBAPS), Barcelona, Spain; 4 Centro de Investigación Biomédica en Red de Salud Mental (CIBERSAM), Madrid, Spain; 5 Departament de Medicina, Universitat de Barcelona, Barcelona, Spain; 6 Division of Psychiatry, Centre for Clinical Brain Sciences, University of Edinburgh, Edinburgh, UK; 7 Generation Scotland, Institute for Genetics and Cancer, University of Edinburgh, Edinburgh, UK

**Keywords:** Prognostic markers, Medical research

## Abstract

Bipolar disorder (BD) involves autonomic nervous system dysfunction, detectable through heart rate variability (HRV). HRV is a promising biomarker, but its dynamics during acute mania or depression episodes are poorly understood. Using a Bayesian approach, we developed a probabilistic model of HRV changes in BD, measured by the natural logarithm of the Root Mean Square of Successive RR interval Differences (lnRMSSD). Patients were assessed three to four times from episode onset to euthymia. Unlike previous studies, which used only two assessments, our model allowed for more accurate tracking of changes. Results showed strong evidence for a positive lnRMSSD change during symptom resolution (95.175% probability of positive direction), though the sample size limited the precision of this effect (95% Highest Density Interval [−0.0366, 0.4706], with a Region of Practical Equivalence: [-0.05; 0.05]). Episode polarity did not significantly influence lnRMSSD changes.

## Introduction

Bipolar disorder (BD) is a severe mental health condition affecting > 1% of the global population^1^. With a population-level annual economic burden estimate of £6.43 billion in the UK alone^2^ and an all-cause mortality rate 1.77 times higher than the general population^3^, BD has huge personal and societal costs. Symptoms encompass disturbances in mood states, thought, energy, and vegetative functions manifesting during episodes of (hypo)mania and depression, the two polarities of BD.

Accumulating evidence^4^ indicates autonomic nervous system dysregulation in BD, detectable through reduced vagally mediated heart rate variability (HRV). This is a measure of the variation in time between consecutive heartbeats and can be computed from interbeat interval (IBI) data collected via either electrocardiogram (ECG) or photoplethysmography (PPG). With the widespread adoption of wearable devices recording IBI data, HRV monitoring can be extended outside the doctor’s office to the patient natural environment, in a near-continuous fashion, unlocking unprecedented opportunities for health monitoring^5^. A number of metrics have been developed to quantify HRV, grouped into time-domain, frequency-domain, and non-linear measures. Among these, the Root Mean Square of Successive RR interval Differences (RMSSD) has been suggested as a robust indicator of vagal tone and parasympathetic activity^6^. RMSSD is indeed the most commonly reported HRV output feature by a number of both commercial^7^ and research-grade devices^8^. Modelling the natural logarithm of RMSSD (lnRMSSD) is common practice, as the log-transformation achieves an easier-to-use, quasi-Gaussian distribution^9–12^.

**Fig. 1:**
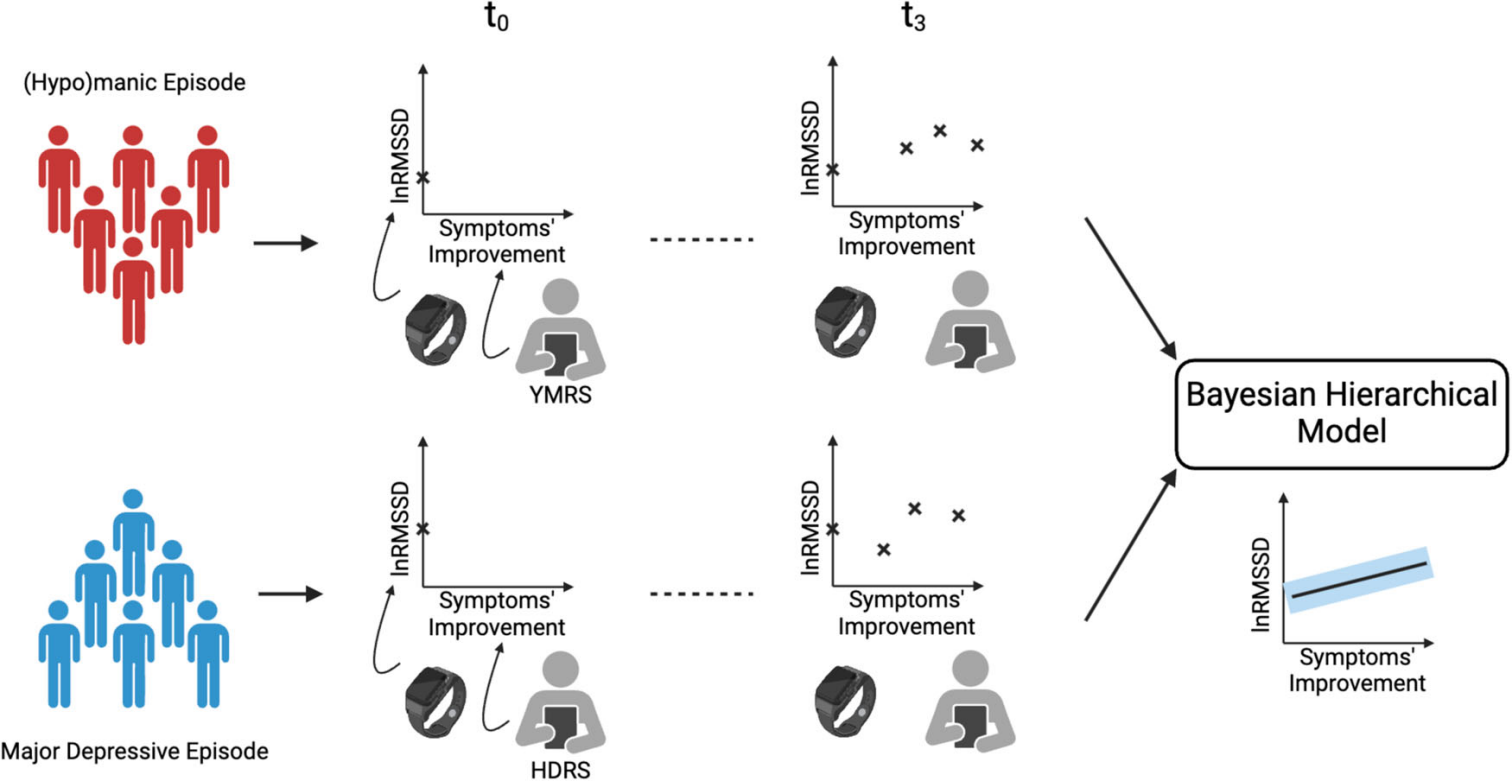
Longitudinal data from patients with bipolar disorder recruited at the onset of an acute episode is used to study the lnRMSSD trajectory as symptoms, as measured with clinician-administered rating scales, improve. Patients with bipolar disorder on either a manic (in red) or a depressive (in blue) episode are assessed up to four times, *t* ∈ {0, 1, 2, 3}, as their symptoms subside. During each assessment, lnRMSSD is collected with a smartwatch while symptoms'improvement is measured by a mental health specialist with a hetero-administered rating scales, the Young Mania Rating Scale^30^ (YMRS) for mania and the Hamilton Depression Rating Scale-17^31^ (HDRS) for depression. A Bayesian Hierarchical Model is fitted to the data to study the rate of change in lnRMSSD with respect to symptoms' improvement. Two models are developed and compared where the only difference is that in one the trajectory of lnRMSSD through symptoms' improvement is allowed to vary across polarities, to test whether a polarity-specific effect on lnRMSSD dynamics exists.

Meta-analyses^13–16^ found a reduced HRV across a range of psychiatric conditions, not just BD, with psychotic disorders featuring the greatest reduction. A reduced HRV is also a predictor of increased cardiovascular risk in the general population^17,18^. As of today it has not yet been fully investigated whether the resolution of symptoms over the course of a BD episode translates into changes in HRV and whether mania and depression, the two polarities of BD, display different HRV trajectories. In this study (Fig. 1) we fill this gap, leveraging the TIMEBASE/INTREPIBD study^19^, a longitudinal cohort following up BD acute episodes.

Studying intra-individual HRV changes across affective states in BD is a challenging and resource-intensive endeavour, especially as longitudinal settings require patients to be followed up and assessed by a mental health specialist multiple times. This is particularly demanding with manic episodes, undermining patients’ compliance to study instructions, such that recruiting large cohorts in HRV studies on BD proves unfeasible and all previous studies had only a couple dozen participants^20–23^.

A case in points is Stautland et al.^20^, limiting their analysis to a sample of 15 patients on a manic episode. A reduced RMSSD in mania relatively to euthymia was found. Participants were assessed only twice – mania and euthymia – and paired two-tailed *t*-tests were used to test zero mean difference across manic and euthymic states. Similarly, Wazen et al.^21^ recruited 19 patients with BD and showed a similar association between RMSSD and mania-to-euthymia transition. Again, only one acute state and one euthymia measurements were taken; a non-parametric (Wilcoxon’s signed-rank) test was used, positing as null a zero median difference between paired observations. On the other hand, Hage et al.^22^ found no significant HRV changes after 8 weeks in 37 patients with bipolar depression randomized to receive either escitalopram-celecoxib or escitalopram-placebo, regardless of treatment response status. The authors opted for a frequency-domain feature, i.e. high frequency (HF-HRV), as their HRV metric and employed repeated measures ANCOVA to evaluate differences between baseline and week 8. Lastly, Faurholt-Jepsen et al.^23^ studied HRV changes in a sample of 16 patients with BD observed for a period of 12 weeks over as many different affective states (euthymia, depression, mania/mixed state) as possible, using a linear mixed-effect model. Investigators found an increased HRV during mania in comparison to both euthymia (in contradiction with^20,21^) and depression, but no significant difference across depression and euthymia. The difference between the second-shortest and the second-longest IBI collected during 30-second epochs was used a HRV measure.

All studies mentioned above^20–23^ collected only one sample per patient per affective state (euthymia, mania/mixed state, depression) and thus did not consider HRV trajectories as a BD acute episode resolves. Moreover, while it is tempting to equate HRV increments/decrements between acute state and euthymia^20–22^ to a process of positive/negative change in HRV, statistical literature^24,25^ warns that two-time points are not sufficient to accurately capture individual differences in trajectories of change and are prone to confounding true change with measurement error. A minimum of three data points per subject is indeed recommended to investigate change over time. Furthermore, as customary in psychiatry research^20–23^, all embraced frequentist null hypothesis significance testing (NHST), failing to propose a model explaining how HRV values are generated and which dependencies among variable govern HRV longitudinal dynamics. Despite its enduring popularity in psychiatry research, the NHST *p*-value has indeed been the object of a growing chorus of criticism^26,27^. The *p*-value serves solely for rejecting the null *H*_0_ and lacks the capacity to assess the extent to which the data supports *H*_0_ versus the alternative hypothesis *H*_1_. Moreover, it measures the existence of an effect but not its magnitude; standardized measures of effect size, since premised on a frequentist framework, inherit its limitations. Further, by simply considering the distribution of a test statistic, previous studies relying on NHST did not elaborate a model trying to capture the data (HRV) generating process.

An alternative framework that has been gaining recognition and popularity in psychiatry research is Bayesian statistics, which mitigates some of the *p* values shortcomings^28,29^. The outputs of Bayesian methods are probability distributions over model parameters, representing the degree of beliefs about parameters’ values, conditional on data and assumptions (the specified model and prior distribution over parameters). Posteriors can be used to make directly interpretable statements about any model parameter of interest, gaining insights into evidence equally for *H*_0_ as for the competing *H*_1_. This is in contrast to frequentist *p*-values, which do not give the probability that a parameter value is compatible with *H*_0_. Bayesian methods are particularly useful with small sample sizes, as it is the case for HRV studies with BD. Indeed, they do not rely on the asymptotic properties of large samples and, thanks to their principled way of handling uncertainty, they yield graded evidence allowing us to gather more information from small studies that may be otherwise underpowered to reach statistical significance. As research into HRV (as well as other digital biomarkers) has the potential for delivering clinical decision support tools, interpretability, i.e. being able to clearly inspect and interrogate the data generating process, and a principled quantification of uncertainty in the model output, are key features of a Bayesian data analysis, that make it particularly appealing in clinical settings.

In this work, using data from the TIMEBASE/INTREPIBD study^19^, we investigate lnRMSSD changes in patients with BD on either mania or depression as their symptoms’ severity, measured with the total score on respectively Young Mania Rating Scale^30^ (YMRS) and Hamilton Depression Rating Scale-17^31^ (HDRS) respectively, wanes, from acute state up to euthymia, with at least three samples available per individual over the course of their episode. Our main contributions are as follows:

- We are the first to the best of our knowledge to study changes in lnRMSSD as an acute episode resolves across both mania and depression within the same cohort.
- We develop an interpretable probabilistic model that captures the natural hierarchical structure in the data (HRV measurements are nested within subjects, subjects on an acute BD episode can be seen as themselves nested within mania and depression) and accounts for how variables interact in generating lnRMSSD. Relatedly, we illustrate the benefits of a Bayesian treatment over NHST, including a principled way to quantify uncertainty and better suitability to small samples than NSHT.
- We fit our model to the data from the TIMEBASE/INTREPIBD study where a minimum of three-time points per individual per affective episode is available. Unlike previous studies only using two-time points (e.g. acute state vs euthymia), this allows us to better capture individual differences in lnRMSSD trajectories. Data does not support the existence of different HRV dynamics across BD polarities, i.e. mania and depression. Results indicate a positive rate of change of lnRMSSD as symptoms’ severity abates from acute episode up to euthymia; however, towards being able to claim that the magnitude of this effect has clinic significance, more data is needed.

## Methods

### The TIMEBASE/INTREPIBD cohort

Unlike other existing cohort, the TIMEBASE/INTREPIBD study^19^ gathers multiple longitudinal assessments per patient over the course of an acute BD episode. This uniquely positions this cohort to investigate trajectories of change in lnRMSSD as an acute episode resolves. TIMEBASE/INTREPIBD is a prospective, exploratory, observational, single-center, longitudinal study with a fully pragmatic design embedded into current real-world clinical practice. A comprehensive description of the data collection campaign is detailed in Anmella et al.^19^. For the purpose of this work, subjects with a DSM-5 diagnosis of BD (equally type I and type II) were considered. Exclusion criteria comprised: concomitant severe cardiovascular or neurological medical conditions with a potential autonomic dysfunction, ongoing cardiovascular arrhythmia, or pacemaker; comorbid current substance use disorder according to the DSM-5 criteria, excluding nicotine substance use disorder; comorbid current psychiatric disorder with great interference of symptoms (e.g., obsessive-compulsive disorder with ritualized behaviours); ongoing pregnancy.

Patients were recruited at the onset of an acute BD episode, either mania or major depression, and were assessed up to four times over the course of their episode: acute phase, clinical response, remission, euthymia (score ≤7 on the HAMD and YMRS for at least 8 weeks^32^). During each assessment, patients were interviewed by a psychiatrist collecting clinical-demographics, including age, sex, medications being administered, and YMRS/HDRS. They were also required to wear the Empatica E4 device^33^ on their non-dominant wrist until battery ran out (~48 hours). This wearable records (sampling rate) 3D acceleration (ACC, 32Hz), blood volume pressure (BVP, 64Hz), electrodermal activity (EDA, 4Hz), heart rate (HR, 1Hz), inter-beat intervals (IBI) and skin temperature (TEMP, 1Hz). Mixed BD episodes were not included in the present analyses in order to minimise diagnostic ambiguity and allow for an easier comparison between the two extreme polarities of BD, also considering that only two such episodes were available in the cohort at the time of this work. Hypomanic episode, on the other hand, were not collected in the TIMEBASE/INTREPIBD study^19^.

### HRV data extraction

During free-living wear, subjects might remove their device or contact to the wrist might be otherwise suboptimal; furthermore, PPG data is affected by motion artefacts, so wake HRV may be unreliable^34^. Thus, we first performed on-/off-body detection using discontinuity in EDA as a guide. In particular, similarly to^35,36^, we considered measurements smaller than 0.05 *μ*S as indicative of off-body status. Then, sleep/wake detection was carried out on on-body recording sequences using the algorithm by Van Hees et al.^37^ which emerged as the best performing in a recent benchmark study on sleep-wake detection^38^.

The RMSSD is arguably the most commonly used HRV metric^7,8^ and reliably captures parasympathetic activity^6^. It is derived from RR intervals (***R***) on either an ECG or a PPG reading and it is computed as follows:

1 $${\rm{RMSSD}}=\sqrt{\frac{1}{N-1}\left(\mathop{\sum }\limits_{i=1}^{N-1}{\left({{\boldsymbol{R}}}_{i+1}-{{\boldsymbol{R}}}_{i}\right)}^{2}\right)}$$

where $\left({{\boldsymbol{R}}}_{i+1}-{{\boldsymbol{R}}}_{i}\right)$ is difference between neighbouring RR intervals and *N* is the total number of RR intervals over which RMSSD is computed. Sleep occurring at nighttime between 10 pm and 5 am from each recording session was segmented with a sliding window of length and step size 5 and 1 minute, respectively, from which RMSSD was derived with FLIRT^39^. This is a popular open-access feature extractor toolkit compatible with E4 data, handling IBI pre-processing and RMSSD computation. The average of all valid 5-minute RMSSD values was taken as a measure for the full night’s RMSSD. This approach to estimate RMSSD is implemented in commercial devices^40^ and was used in previous research^41^. Five minutes is indeed a conventional length for RMSSD estimation^6^. Considering motion artefacts and circadian rhythms in HRV, nighttime sleep is a popular choice for HRV extraction; averaging over multiple 5-minute RMSSD is more robust than using just a random 5-minute RMSSD which would be susceptible to HRV variations across sleep stages^42^. Recording sessions from the TIMEBASE/INTREPIBD study stretched over 48 hours so, while two nights were available for HRV extraction, only the first one was considered, since closer to the time when HDRS/YMRS were taken. As standard practice^9–12^, we modelled lnRMSSD, that is the natural logarithm of RMSSD, as this transformation results in an more convenient, quasi-Gaussian distribution. While wristbands today allow for collecting RMSSD, they do not provide a model explaining how features of the individual interact in generating RMSSD values. In the section that follows, we build a Bayesian model attempting to do just that.

### Bayesian modeling

The goal of inference is to get to unobserved parameters (*Θ*), given the data. The Bayesian approach aims for a full distribution over *Θ*, not just a single value, which, especially when data is scarce, can be misleading, since it does not consider uncertainty and tells only a part of the story (e.g. the mean or the mode of the distribution). Our Bayesian analysis is particularly interested into the rate of change of lnRMSSD with respect to symptoms’ severity, so this will be a key parameter of interest. The Bayesian paradigm commands to posit a process generating the data at hand governed by *Θ*, referred to as likelihood *P*(Data∣*Θ*), as well as a starting hypothesis as to what values *Θ* can credibly take, in advance of seeing any data, referred to as the prior *P*(*Θ*). The output of Bayesian inference is a posterior *P*(*Θ*∣Data), where the prior beliefs about the values of *Θ* have been updated in light of the observed data.

As a running example to illustrate Bayesian methods, we temporarily assume here that the observed lnRMSSD values are sampled from a Gaussian distribution with mean *μ* and variance *σ*^2^, the latter we assume given and equal to 1. As with ordinary regression, parameters can be modelled as a function of relevant covariates. For example, we might have reasons to believe that *μ* linearly depends on the symptoms’ severity (*V*) of the individuals: *μ* = *θ*_0_ + *θ*_1_*V*_*i*_, where *i* indexes the subjects in the study. The parameters of our model are thus *θ*_0_ and *θ*_1_ and our interest might be into *θ*_1_, expressing the dependency of lnRMSSD on *V*. The likelihood *P*(Data∣*Θ*) is a function of the parameters, expressing the probability of observing the given data under particular values of *Θ*, in our example, how well different values of *θ*_0_ and *θ*_1_ explain the data.

The other key ingredient of a Bayesian model, further to the likelihood, is the prior probability over the parameters *P*(*Θ*), representing our beliefs about the parameters before seeing any data. The choice of prior can be informed by previous research. Alternatively, in case of lack of previous evidence or when the analyst does not want to favour one hypothesis over others, a non-committal prior can used, assigning equal credibility to competing hypotheses. In the running example we might opt for ${\theta }_{0} \sim {\mathcal{N}}\left(0,1\right)$ and ${\theta }_{0} \sim {\mathcal{U}}\left(-1,1\right)$, i.e. a standard Gaussian for the intercept *θ*_0_, favouring values around zero but not giving any preference to either positive or negative values, and a uniform distribution for the slope *θ*_1_, assigning equal credibility to all values in the interval [-1,1].

Through Bayes’ theorem, the prior is updated in light of the observed data to yield a posterior probability distribution *P*(*Θ*∣Data): this encapsulates the refined beliefs about the parameters, incorporating both prior knowledge and the information conveyed by the observed data. In our example, we might move from a flat prior over *θ*_1_ to a distribution where the overwhelming majority of the probability density is concentrated on positive values. This posterior, *P*(*θ*_1_∣Data), can be directly and naturally interpreted as our beliefs about values of *θ*_1_, condition on the observed data and the posited model. This is arguably more intuitive for clinicians to use than a *p*-value, the probability of obtaining under the null hypothesis (*H*_0_) and under the assumed sampling intention a result equal to or more extreme than the one observed from the data, and can be directly used to make statements about both the existence and the magnitude of an effect.

The only extra layer of complexity in Bayesian hierarchical models, on top of the vanilla Bayesian machinery we introduced above, is that parameters depend on other parameters too, referred to as hyperparameters, introducing dependencies between parameters at different hierarchical levels. This is particularly convenient as it allows us to model lnRMSSD observations as nested into subjects and subjects themselves as nested into episode polarity *π*. In our running example, we can modify the model to reflect that the relation between *V* and lnRMSSD might differ across polarities as follows: ${\theta }_{1} \sim {\mathcal{N}}\left({\zeta }_{\pi [i]},1\right)$ and ${\zeta }_{\pi } \sim {\mathcal{U}}(-1,1)$. This is now saying that the intercept *θ*_1_ is sampled from a Gaussian whose mean is controlled by another parameter *ζ*_*π*_ with a uniform prior on [-1,1]. There are *Π* parameters *ζ*, one for each polarity and all sampled from the same uniform distribution. The notation *π*[*i*] denotes the parameter *ζ* that corresponds to the polarity *π* to which the *i*^*th*^ individual’s episode belongs to. It can be seen how hierarchical models provide a powerful framework for nested data: in our study, each patient (level-1) generates multiple lnRMSSD measures since patients are indeed assessed at multiple time points as their symptomatology improves; secondly, from each BD polarity (level-2) multiple patients are drawn. Hyperparameters enables sharing of information across level groups, while allowing for within-group variability. Conceptually, a hierarchical model provides a middle ground (*partial pooling*) between aggregating groups at a given level of the hierarchy (*complete pooling*), thus overlooking potential differences across groups, and treating them as completely independent (*no pooling*).

### Variables preprocessing

We wanted to model how lnRMSSD changes as symptoms’severity, measured with the total score on either YMRS (manic episode) or HDRS (depressive episode), abates during the resolution of an acute BD episode. Each *i*^*th*^ individual of the *N* included in the analyses was sampled up to four times along their trajectory of symptoms’ improvement, starting from episode onset *t* = 0. For the *i*^*th*^ individual, their improvement along this trajectory at time *t* ∈ {0, 1, 2, 3} was expressed as ${I}_{i,t}=({{\rm{score}}}_{\pi [i],t = 0}-{{\rm{score}}}_{\pi [i],t})/({{\rm{score}}}_{\pi [i],t = 0})$, where the notation *π*[*i*] means that the total score on YMRS (HDRS) was used if the episode’s polarity *π* of the *i*^*th*^ individual was manic (depressive). *I* therefore takes values in [0, 1], patients have a value of 0 at episode onset, i.e. study recruitment, and reach a value of 1 if their total score goes down to 0; intermediary values express fractional improvement with respect to episode’s onset severity. For a given subject, successive recording sessions were required to have a strictly monotonic decrease in the relevant scale’s total score.

A number of factors further to changes in symptoms’ severity can influence HRV. We therefore controlled for relevant covariates available in our dataset, i.e. sex *S* (females = 1, males = 0), age *A*, and medications *M*. Age (in years) was standardized and treated as constant across different recording sessions for a given individual. Data for a number of drug classes known to affect HRV was recorded in the INTREPIBD/TIMEBASE dataset as boolean: lithium, selective serotonin reuptake inhibitors, serotonin and norepinephrine reuptake inhibitors, tricyclics, monoamine oxidase inhibitors, other antidepressants, typical antipsychotic, atypical antipsychotic, anticonvulsants, beta-blockers, opioids, amphetamines, antihistamines, antiarrhythmic agents, other anticholinergic medications, benzodiazepines. *M*_*i*,*t*_ is simply the total number of such medications the *i*^*th*^ individual was on at time *t*. Lastly, as previous research in cross-sectional samples suggested that HRV is negatively correlated with symptoms’ severity^43^, we accounted for baseline severity ${B}_{i}={{\rm{score}}}_{\pi [i],t = 0}/\max (q)$ where the denominator is the maximum value by design on either the YMRS or HDRS rating scale, depending on whether the episode’s polarity of the *i*^*th*^ subject was mania or depression.

### Regression models

**Fig. 2:**
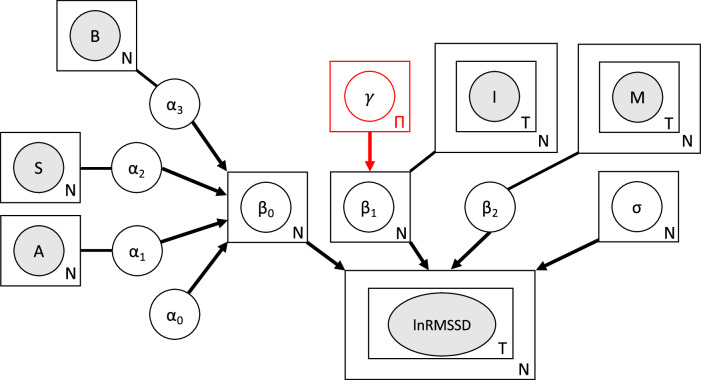
lnRMSSD data generating process assumed in the regression models. Grey-shaded nodes represent observed variables, while white nodes represent the model’s parameters. Arrows define conditional dependencies in the model graph, while lines connecting parameters to their covariates do not define any probabilistic dependency but are shown simply to clarify which covariate a parameter refers to. The plate notation is used for observed variables and parameters that are repeated, where the letter indicates the number of repetitions; in other words, it indicates the nested structure in the data and in the model. For example, lnRMSSD is contained in two plates: the outer one indicating that samples are drawn at the subjects' level where *N* is the total number of subjects, the inner one indicating that within each of the *N* individuals, samples are taken at *T* times. The node for *γ* and its outgoing arrow are in red to mark that this node, and thus the dependency of its descendants on episode’s polarity where there are *Π* = 2 polarities (mania and depression), is only present in the *two-polarities-model* into which the *one-disease-model*, differing only by the lack of this node, is nested. A: age; S: sex; B: baseline symptoms' severity; I: symptoms' improvement; M: medications.

We developed two hierarchical linear models, which we nicknamed *two-polarities-model* and *one-disease-model*, illustrated in Fig. 2, where the only difference is that the former allows the lnRMSSD rate of change with respect to symptoms’ improvement to vary across polarities (manic and depressive), letting us test whether a specific polarity effect is supported by the data.

In the *two-polarities-model*, we assumed that lnRMSSD for the *i*^*th*^ subject at time *t* is drawn from a Gaussian ${\mathcal{N}}$ whose mean is a linear combination of the intercept *β*_0,*i*_, symptoms’ improvement *I*_*i*,*t*_, and medications *M*_*i*,*t*_:

2 $${{\rm{lnRMSSD}}}_{i,t} \sim {\mathcal{N}}\left({\beta }_{0,i}+{\beta }_{1,i}{I}_{i,t}+{\beta }_{2}{M}_{i,t},{\sigma }_{i}\right)$$

The subscripts denote that while *β*_2_ does not vary across either individuals or time, each individual has their own intercept term *β*_0,*i*_ and coefficient *β*_1,*i*_. This allows each individual to have their own intercept and rate of change with respect to *I* but crucially these parameters are drawn from a common distribution, as shown below. As regards *β*_0,*i*_, i.e. the expected value lnRMSSD takes when *I*_*i*,*t*_ = 0 (episode onset) and *M*_*i*,*t*_ = 0 (no medications with a known effect on HRV), we modelled it as drawn from a Gaussian with a standard deviation fixed to 0.5 but whose mean linearly depends on sex *S*_*i*_, age *A*_*i*_, baseline severity *B*_*i*_ plus the intercept *α*_0_:

3 $${\beta }_{0,i} \sim {\mathcal{N}}\left({\alpha }_{0}+{\alpha }_{1}{A}_{i}+{\alpha }_{2}{S}_{i}+{\alpha }_{3}{B}_{i},0.5\right)$$

As for *β*_1,*i*_, i.e. the rate of change of lnRMSSD with respect to symptoms’ improvement, subjects on different episode polarities draw their slope *β*_1,*i*_ from Gaussian distributions centred at different values:

4 $${\beta }_{1,i} \sim {\mathcal{N}}({\gamma }_{\pi [i]},0.1)$$

Here *π*[*i*] indeed signifies the mean *γ* corresponding to the group (polarity *π*) to which the *i*^*th*^ individual’s ongoing episode belongs. We defined subject-specific lnRMSSD standard deviation *σ*_*i*_ as drawn from an inverse gamma distribution. The inverse gamma distribution is a convenient choice here, as it is the conjugate prior of a normal distribution with unknown mean and variance. Conjugacy speeds up inference by enabling a closed-form solution to (part of) the posterior:

5 $${\sigma }_{i} \sim {\mathcal{IG}}\left(3,0.5\right)$$

The prior for *α*_0_ is a Gaussian centred at the sample average lnRMSSD, i.e. ${\overline{\mu }}_{lnRMSSD}$:

6 $${\alpha }_{0} \sim {\mathcal{N}}\left({\overline{\mu }}_{lnRMSSD},0.1\right)$$

*α*1, *α*2, *α*3, and *β*_2_ all had a Gaussian prior with mean -0.1 and standard deviation 0.1, informed by previous research showing that female sex, older age, greater symptoms’ severity at onset, and the medications mentioned above are associated with a lower HRV^43–45^:

7 $${\alpha }_{0},{\alpha }_{1},{\alpha }_{2},{\beta }_{2} \sim {\mathcal{N}}\left(-0.1,0.1\right)$$

On the other hand, we made a non-committal choice for the prior over *γ*_*π*_, i.e. a uniform distribution assigning equal probability density to values in the zero-centered interval [-1, 1]:

8 $${\gamma }_{\pi } \sim {\mathcal{U}}\left(-1,1\right)$$

In other words, we start from a sceptical position and in advance of seeing any data we do not favour any value for the polarity-specific mean of the Gaussian from which *β*_1,*i*_ is drawn.

The *one-disease-model* only differs by the lack of dependency of *β*_1,*i*_ on the episode’s polarity. Here, the prior on *β*_1,*i*_ is a non-committal uniform:

9 $${\beta }_{1,i} \sim {\mathcal{U}}\left(-1,1\right)$$

Consequently, the *one-disease-model* pools subjects together regardless of polarity but, as with the *two-polarities-model*, *β*_0,*i*_ and *β*_1,*i*_ can still vary across subjects while being sampled from the same distribution.

There are different approaches to Bayesian inference. For example, simple models relying on exponential family distributions and conjugacy admit analytical solutions. Often times, however, with more complex models, as it is the case with our hierarchical models, different approaches are required, e.g. sampling-based solutions or variational inference. We adopted the Hamiltonian Monte Carlo (HMC) No-U-Turn Sampler (NUTS)^46^, as state of the art inference algorithm and default choice across a number of probabilistic programming libraries^47,48^. In particular, we ran four parallel chains of 2000 tuning steps, 2000 samples, and a target acceptance probability of 0.99 was used for Bayesian inference in both models.

As explained above, the *two-polarities-model* and *one-disease-model* encapsulate different assumptions about the data-generating process. In particular, the former allows the rate of change of lnRMSSD with respect to symptoms’ severity to vary across episode’s polarity, while the latter does not account for episode polarity. Towards model comparison, i.e. to assess which of the two models betters explains our data, we used the Widely Applicable Bayesian Information Criterion (WAIC)^49^. WAIC calculates an estimate of the out-of-sample log-likelihood and adjusts for the effective number of parameters, providing a more accurate measure of a model’s fit and predictive ability. The value of WAIC lacks inherent meaning and only becomes meaningful when comparing it across different models fitted to the same data. Lower WAIC values suggest a better fit of the model to the data. We chose WAIC over other criteria for its Bayesian consistency, effectiveness with complex models, incorporation of uncertainty, focus on predictive accuracy, applicability to hierarchical structures, and bias correction, offering a robust approach. The Bayesian factor, comparing model likelihoods based on observed data, is another tool for selecting between models but faces criticism for its sensitivity to the prior specification, even when different priors lead to minor differences in the posterior^50^.

We plotted samples from the posterior distributions over the parameter(s) relevant to our investigation into RMSSD changes with respect to symptoms’ improvement (potentially varying across polarities). Towards summarizing the posterior, we computed the Probability of Direction (PD)^51^. This is an index of effect existence, robust to the scale of both the response variable and the predictors. It ranges from 50% to 100%, representing the certainty with which an effect goes in a particular direction (i.e., is positive or negative), and is mathematically defined as the proportion of the posterior distribution that is of the median’s sign. We also computed the 95% highest density interval (HDI-95), i.e. the 95% most plausible values in a parameter’s posterior. This is more suited than the PD to measure the magnitude of an effect by comparing its overlap with a Regional of Practical Equivalence (ROPE); this is a range of values considered negligible or too small to be of any practical relevance for the use case in question^51,52^. Unlike PD, HDI and ROPE are sensitive to the parameter’s scale. For the posterior over *β*_1_, obtained by pooling together samples from all individuals’ *β*_1,*i*_ to study the overall effect across individuals, we set a ROPE of [-0.05, 0.05]. As we are modelling lnRMSSD, for a given sample ${\hat{\beta }}_{1,i}$ of *β*_1,*i*_ a unit change in *I*_*i*,*t*_ (i.e., 100% improvement in symptoms over baseline severity) translates into a change of ${\hat{\beta }}_{1,i}$ in lnRMSSD for fixed values of other predictors in Equation (2). This is the standard interpretation of regression coefficients. When mapping back onto the original scale of RMSSD, if ${\hat{\beta }}_{1,i}$ equals an arbitrary value *c*, RMSSD changes with respect to its baseline value by a multiplicate factor of *e*^*c*^, where *e* is the base of the natural logarithm. In fact, by the properties of logarithms, if *ln*(*Y*_*t*=*T*_) − *ln*(*Y*_*t*=0_) = *c*, then *Y*_*t*=*T*_ = *Y*_*t*=0_ × *e*^*c*^ for any arbitrary *c*. Thus, the ROPE of our choice considers negligible any multiplicate effect of a complete resolution of symptoms on RMSSD between *e*^−0.05^ = 0.951 and *e*^0.05^ = 1.051, in other words, a decrease (increase) of 4.9% (5.1%).

### Ethical approval statement

The TIMEBASE/INTREPIBD study was conducted in accordance with the ethical principles of the Declaration of Helsinki and Good Clinical Practice and the Hospital Clinic Ethics and Research Board (HCB/2021/104). All participants provided written informed consent prior to their inclusion in the study. All data were collected anonymously and stored encrypted in servers complying with all General Data Protection Regulation regulations.

## Results

### Study sample

At the time of this study, a total of 67 patients with BD had been recruited at the onset of a mood episode (29 depression, 38 mania) in the TIMEBASE/INTREPIBD study. Ultimately, a sample of 23 patients were available for this study: 41 dropped out before providing a minimum of three assessments, while 3 did not have a strictly monotonic decrease in their symptoms’ severity, thus preventing the use of improvement on symptoms’ severity to clock time in our model of change. 9 (resp. 14) individuals were recruited at the onset of a major depressive (resp. manic) episode. 17 (resp. 6) subjects had 3 (resp. 4) follow-up assessments. The median (resp. interquartile range) time (in years) since illness onset was 5 (resp. 17.5). Clinical-demographics are given in Table 1. Figures for the sleep time during the 10 pm to 5 am interval from which RMSSD was extracted are given in Supplementary Table 1. The median percentage of 5-minute sliding windows over sleep time not passing quality control with FLIRT, thus outputting a nan value, was 9.05 (interquartile range 1.95-25.32). Such segments were discarded from analyses and thus not considered in the computation of the night RMSSD.

**Table 1:** Clinical-demographic features of the study sample

	Age	Females	Medications #	Baseline symptoms’ severity
	mean (std)	N (percentage)	mean (std)	mean (std)
**Mania**	42.14 (12.81)	5 (35.71%)	2.86 (1.30)	YMRS
N=14				25.64 (5.09)
**Depression**	44.34.56 (13.03)	6 (66.67%)	3.78 (0.63)	HDRS
N=9				19.11 (3.21)

“Medications #” refers to the number of drugs recorded in our cohort with a known influence on HRV, which subjects were taking at the moment of study admittance; further details on medications are given in Supplementary Table 2. We report clinical-demographic features for the 44 patients not included in the present analyses as not providing a minimum of three HRV samples in Supplementary Table 3. Total score on Young Mania Rating Scale (YMRS) and Hamilton Depression Rating Scale-17 (HDRS) was used to track symptoms’ severity in manic and depressive episodes, respectively. The figures herewith shown refer to the first assessment (acute episode onset). Note that, as YMRS and HDRS do not share the same range ([0-60] and [0-52], respectively), the percentage of improvement with respect to onset total score was used to clock time across polarities in the regression model.

### Prior predictive checks

As customary in a Bayesian data analysis, before model fitting, we ran a series of checks, referred to as prior predictive checks, whose purpose is to assess the soundness of the model assumptions. This is particularly useful in hierarchical models, where the effect of hyperparameters might propagate downstream in the data-generating process in hard-to-predict ways. Specifically, we verified that, as desirable, in advance of seeing any data the implied distribution over lnRMSSD, i.e. the distribution obtained sampling from the model prior and generating synthetic lnRMSSD values, covered the sample distribution of lnRMSSD and had the bulk of the density lying within physiologically plausible values. Secondly, we verified that, before seeing the data, the model did not favour either positive or negative values for the lnRMSSD rate of change with respect to symptoms’ improvement.

**Fig. 3:**
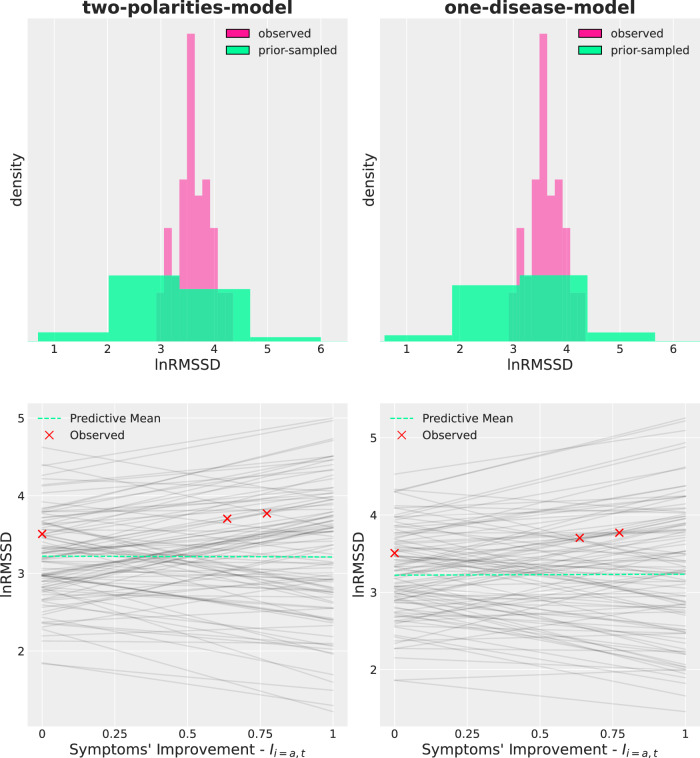
Prior predictive checks across the two regression models. The left column refers to the *two-polarities-model* while the right column to the *one-disease-model*. The (normalized) histograms in the top row show the observed lnRMSSD distribution against the lnRMSSD distribution implied by the prior. It can be seen that the observed lnRMSSD (pink) is tightly concentrated over a narrow range in comparison to the prior lnRMSSD (green), which puts some probability density on values at the boundaries of the physiologically plausible range. However, the bulk of the prior lnRMSSD contains the observed lnRMSSD. The three red crosses in each bottom row plot shows lnRMSSD measures at different stages of symptoms' improvement for a subject from our dataset, chosen as a way of example and assigned the dummy subject-id *a*. Superimposed are one hundred lines, each showing the expected lnRMSSD value for different draws from the prior. As a result of a vague and non-committal prior, lines can have a variety of slopes with no preference for either positive or negative values. The dashed green line represents the average across the one hundred black lines.

The top row of Fig. 3 shows the prior distribution over lnRMSSD across both the *two-polarities-model* (left) and the *one-disease-model* (right) against the one observed in the data. The two models have similar prior lnRMSSD distributions, which contain the observed data. However, probability is spread over a range of lnRMSSD values slightly broader than the one in the data, whist still keeping within physiologically plausible values. The 0.05, 0.5, and 0.95 quantiles (*q*_0.05_, *q*_0.50_, *q*_0.95_) were respectively 1.88, 3.21, and 4.51 (1.89, 3.21, and 4.50) for the *two-polarities-model* (*one-disease-model*). The Kullback-Leibler divergence for the prior distribution over lnRMSSD from the *two-polarities-model* to the *one-disease-model*, a measure of “distance” between distributions taking values in [0, + *∞*], was 0.00006. On the other hand, *q*_0.05_, *q*_0.50_, *q*_0.95_ were respectively 3.08, 3.60, and 4.18 for the sample lnRMSSD.

The bottom row of Fig. 3 shows the implied distribution over lines within a subject (shown as a way of example), each line representing a hypothesis, i.e. a sample from the prior, about the expected lnRMSSD value as a function of symptoms’ improvement upon onset severity. In both models the subject’s true values lie with the array of lines in both model, the lines’ origin is centred roughly around the sample average lnRMSSD and, as a result of the non-informative prior, a broad range of slopes is credible under the prior with no preference for either positive or negative values (positive or negative rate of change of lnRMSSD with respect to symptoms’ improvement).

### Model convergence and comparison

In order to infer the posterior distribution over the model parameters, we resorted to Markov Chain Monte Carlo (MCMC) methods, in particular NUTS^46^, as our models did not admit an exact, closed-form solution. MCMC involves generating a sequence of random samples, known as chains, which approximate the posterior distribution. However, convergence to the true posterior distribution is not guaranteed, so it’s crucial to assess the convergence and mixing properties of the chains. This is typically done using diagnostics such as the Effective Sample Size (ESS), Gelman-Rubin convergence diagnostic ($\hat{R}$), and Bayesian Fractions of Missing Information (BFMIs). In both the *two-polarities-model* and the *one-disease-model* the chains mixed well with all ESS > 1000, all $\hat{R}=1$, and all BFMIs ≥0.75.

The WAIS for the *two-polarities-model* and the *one-disease-model* was respectively -92.94 and -98.90, indicating that, conditional on our data, the latter model, not positing the lnRMSSD rate of change with respect to symptoms’ improvement as dependent on the episode’s polarity, is a better fit.

### lnRMSSD rate of change with respect to symptoms’ improvement

Further to investigating possible differences across the episode’s polarities, a central question in our investigation was how lnRMSSD changed across the trajectory of symptoms’ improvement, from episode onset up to euthymia. As the *one-disease-model* came out on top in model comparison, we collected and pulled together posterior samples from *β*_1,*i*_ across the *N* = 23 individuals in our analyses, in order to study the overall effect *β*_1_ regardless of the specific subject.

**Fig. 4:**
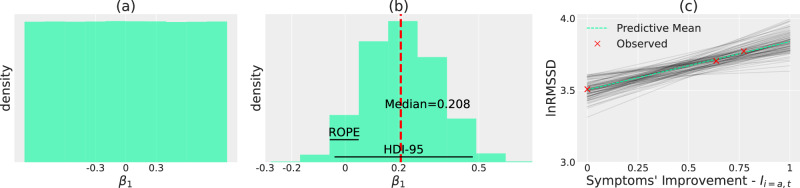
Prior and Posterior distributions over *β*_1_. **a**: prior distribution over *β*_1_. **b**: posterior distribution over *β*_1_ along with median (red, dashed line), 95% Highest Density Interval (HDI-95) spanning [-0.03662-0.47061] and Region of Practical Equivalence (ROPE) at [-0.05, 0.05]. **c**: posterior distribution over expected lnRMSSD values as a function of symptoms' improvement for a subject recruited at the onset of a manic episode, identified with the dummy subject-id *a*. The posterior for the other subjects is available in Supplementary Fig. 2. Each black line (a total of one hundred is herewith displayed to avoid clutter) represents a single draw from the posterior, while the dashed green line is the average across all black lines sampled from the posterior. This illustrates how the Bayesian framework naturally incorporates uncertainty in its outputs, as in this plot we indeed have a distribution over lines and not just a single line. This notion of uncertainty enables better-informed decisions in a clinical setting, e.g. the confidence in a given positive trend in lnRMSSD is higher when lines are tightly packed around the average value.

Figure 4 a illustrates the prior distribution, defined in Equation (9), for *β*_1_. It can be seen how the prior is non-committal and vague, as it does not favour any value in the interval [-1,1] and admits a broad variability in the effect that *I*_*i*,*t*_ can have on lnRMSSD, from -1 to 1 (the scale is logarithmic).

Figure 4b illustrates the posterior distribution over *β*_1_. Bayesian inference reassigned credibility so that relatively strong effects of *β*_1_ on lnRMSSD have very little probability densities, i.e. values below (above) -0.5 (0.5), while hypotheses compatible with the data now have higher density. Contrast how, upon conditioning on the data, the distribution on *β*_1_ changed from Fig. 4a to b. We calculated commonly used statistics and decision rules on the posterior. The median (dashed red line) lies at 0.208. The PD indicates that *β*_1_ is strictly positive with high probability, i.e. 95.175%. It can indeed be seen that samples from the posterior overwhelmingly favour positive values. The HDI-95, i.e. the narrowest interval containing 95% of the posterior probability density, spans [-0.03662–0.47061], thus overlapping but not containing the rope [-0.05, 0.05]. As per^52^ recommendations, the HDI-95-based decision rule is therefore to withhold decision and collect more data to increase the precision of the estimates.

Figure 4 c lastly shows the posterior for the same individual reported in prior predictive checks, bottom-right of Fig. 3, to whom the dummy identifier *a* was assigned. The distribution over lines now span only a narrow range of possible values, with a tendency for positive values. The posterior distribution for other subjects in the study can be seen in Supplementary Fig. 2 and overall confirms the positive trend in *β*_1_ values.

The posterior over the co-variates’ coefficients, i.e. age, sex, onset symptoms’ severity, and number of medications with an influence on HRV, can also been seen in Supplementary Fig. 5. In general, the posterior did not differ much from the prior distribution in either shape or direction; however, for *β*_2_, i.e. the coefficient associated with the number of medications known to affect HRV, the posterior sharpened and its HDI-95 excluded the 0 value.

## Discussion

In this work, we studied how lnRMSSD changes as the symptoms’ severity subsides over the course of an acute BD episode. Our findings do not support a specific effect of polarity, i.e. mania or depression, on the dynamics of change in lnRMSSD. To the best of our knowledge, only the work by Faurholt-Jepsen et al.^23^ considered HRV across the full BD spectrum but only took one HRV sample per episode across patients, thus not investigating within-episode dynamics and limiting comparability with this study. The lack of a polarity-specific component to HRV trajectories in our study suggests that within-episode HRV changes may not be useful to distinguish between manic and depressive phases. On the other hand, our findings support with high confidence the existence of a positive rate of change of lnRMSSD with respect to symptoms’ improvement over the course of an acute BD episode. However, our data did not show that the HDI-95 completely excludes the ROPE. This is likely related to the sample size, as sensitivity analyses (Supplementary Note 1) showed that increasing either the number of recruited subjects or the number of observations per subject led to a higher chance of a model fit where the HDI-95 completely excludes the ROPE, assuming a data generating process where the HDI-95 on the distribution for the lnRMSSD slope (*β*_1_) does exclude the ROPE. While the Bayesian approach commands to consider the entire distribution, the HDI-95 summary and the ROPE-partial-overlap rule^52^ suggests withholding decision and collect more data before developing an intervention that might depend on the parameter of interest completely excluding the ROPE.

Sample size is indeed a limitation of this and previous studies into intraindividual HRV changes in BD, since collecting longitudinal data from patients with BD, especially when on a manic episode, is a resource-intensive endeavour. The inherent limitation of sample size hinders the frequentist approach^53^ used in previous studies. We thus opted for a Bayesian approach in our work, as it is more suitable to small samples and capable of quantifying uncertainty in a principled manner, a desirably property when data is used to inform decision-making in potentially high-risk environments such as healthcare. Furthermore, we went beyond simply assessing the distribution of a test statistic and proposed an explainable probabilistic model that attempts to explain how lnRMSSD values are generated across successive observations within-subjects and how different clinical-demographic covariates interact in this process.

Consistently with our results, the majority of previous studies investigating intra-individual HRV changes from mania to euthymia, while only collecting two samples per patient, found a positive difference^20,21^. Previous cross-sectional studies comparing patients on a manic episode to healthy controls also found a reduced HRV in mania^54^. Of importance, HRV in euthymic BD remains lower than in healthy controls despite full clinical remission, even though at least part of this difference is likely due to medications^55^. As regards studies into bipolar depression, one^22^, taking only a sample from acute state and one from euthymia, did not find any significant difference in HRV across acute state and euthymia. However, a cross-sectional study^43^ found a negative association between symptoms’ severity and HRV. The inconsistency of findings in the literature may in part be a result of the sample size used in this type of studies and the frequentist approach. The Bayesian approach we herewith adopted is arguably better suited as it yields graded evidence, suggesting when collecting more data is likely to be fruitful. Secondly, we note that studies differ in the HRV metrics they employed and, more importantly, the device used for IBI data collection and the algorithms for IBI pre-processing. This could also explain inconsistency in findings. For the sake of transparency and reproducibility, we release the codebase we developed for these analyses.

The results of this study need to be balanced against some limitations. 1) We could not include BMI, alcohol, and nicotine intake as covariates in our models since these HRV confounders were not collected in the TIMEBASE/INTREPIBD study. Similarly, while unlike some previous studies (e.g.^16^) we included medications, we did not account for their plasma concentration, receptor profile, or interactions but only considered the total number of known interfering drugs. 2) We took one step beyond previous studies and fitted a model of change with at least three samples available per subject per episode, however the lack of a higher number of intra-individual observations constrained us to fit a linear model since non-linear patterns may not be identifiable with only three data points. However, we do not have reasons to exclude a non-linear trajectory. 3) The limited sample size likely prevented us from asserting the magnitude of the rate of change in lnRMSSD with respect to symptoms’ improvement in a way to exclude a region of practical equivalence, and further research in this sense is needed.

In conclusion, previous converging evidence indicated an HRV reduction in BD relatively to healthy controls, pointing to an impairment in the autonomous nervous system. This study, the first to the best of our knowledge to include a minimum of three observations per patient per episode across both polarities of BD, suggests that an improvement in symptoms’ severity upon an acute episode is paralleled by a positive change in HRV. However, the pattern of HRV change does differ across mania and depression, the two polarities of BD. Thus, our findings suggest that HRV, thanks to an increasing adoption of wearable devices, may have a role in monitoring the course of an episode in clinical settings, acting as a measurable biological signal, which can complement clinical assessments; however, it may be not useful towards distinguishing polarities in BD. Studies of HRV in BD have been dogged by limited sample size, a limitation inherent to this type of studies. Crucially, unlike frequentist statitics, the Bayesian framework we herewith adopted, allowed for a fine-grained appreciation of the evidence, inspecting posterior distributions conditioned on the data (and the posited model), and the formulation of a generative, interpretable probabilistic model accounting for how different variables interact in generating HRV values within patients over the course of a BD episode.

## Supplementary information

Supplementary Information

## Data Availability

The data used for the present study can be made available through reasonable requests to the corresponding author due to data sharing restrictions

## Code availability

The codebase developed for this work is available at https://github.com/april-tools/bayesian-hrv. Python 3.10 programming language was used, with Bayesian statistical modelling implemented in PyMC^48^ and ArviZ^56^.
