# Supplementary material for: A Bayesian analysis of heart rate variability changes over acute episodes of bipolar disorder
Source: Npj Ment Health Res. 2024 Oct 3;3:44. doi: 10.1038/s44184-024-00090-x (PMC11449927; doi:10.1038/s44184-024-00090-x)
Supplement: Supplementary file 1 — Supplementary Information [file 44184_2024_90_MOESM1_ESM.pdf]

Supplementary information

Supplementary Note 1 Sensitivity analyses

In sensitivity analyses, first (**i**), we assessed to what extent the results from the *one-disease-model* were influenced by the choice of prior for $\beta_{1,i}$. Second (**ii**), we examined with what frequency the HDI-95 would completely exclude the ROPE if multiple synthetic datasets were generated according to the *one-disease-model*, sampling $\beta_{1,i}$ from $\mathcal{N}(0.247, 0.1)$ – in other words positing for the data generating process a distribution on $\beta_{1,i}$ whose HDI-95 does exclude the ROPE [-0.05, 0.05] – and the *one-disease-model* described in was fit to each such dataset. This experiment indicates how likely our pipeline is to recover a distribution whose HDI-95 does not overlap with the ROPE, assuming the true data generating process is indeed governed by such a distribution, under the constraints of a limited sample size (N=23) with up to four follow-up measurements per patient. We also investigated how increasing either the number of follow-up observations per patients or the sample size would affect the chances of recovering a posterior whose HDI-95 excludes the ROPE.

i We experimented with two alternative choices of priors for $\beta_{1,i}$ in the *one-disease-model*: (a) a beta distribution with parameters a and b of 5 and 2, scaled by 1.5 and shifted by -0.85, and (b) a normal distribution with parameters μ and σ of 0 and 0.1. The probability density function (PDF) of the two distributions is displayed in Supplementary Figure 1. (a) is a distribution favouring positive values for $\beta_{1,i}$, the area under the curve (AUC) to the right of 0 is indeed 81.50%. On the other hand, (c) has its mode at zero and does not favour positive over negative values or vice versa, the AUC between -0.1 and 0.1 is 68.26%. (a) and (b) have a positive probability of direction of 96.677% and 95.152% respectively, but neither led to an HDI-95 excluding the ROPE ([-0.0120, 0.4658] and [-0.0952, 0.2500] respectively). The WAIC was -92.623 and -91.992 respectively.

ii Synthetic data for age was generated from a Gaussian whose mean and standard deviation were set to the sample mean and standard deviation. Sex was sampled from a Bernoulli with mean set to the sample proportion of female participants. Baseline severity was sampled from a uniform, with support going from the sample minimum baseline severity to 1. Medications's number was sampled from a discrete uniform distribution over [2, 3, 4, 5, 6] where the probability for a patient of remaining on the same number of medications was 97.5%. This value reflects the clinical practice tendency to use continue the starting treatment regime throughout the episode. 73.91% (26.09%) of the subjects were sampled three (four) times over their trajectory of symptoms' improvement, where the first sampled was collected at 0, i.e. acute episode onset, as per the study design. Other observations were sampled uniformly at random at a symptoms' improvement position between 0.2 and 1. 73.91% (26.09%) corresponded to the fraction of patients in our sample with three (four) observations. All parameters but $\beta_{1,i}$ were sampled according to prior specified in . For $\beta_{1,i}$ we assumed a normal prior $\mathcal{N} \sim (0.25, 0.1)$. As shown in Supplementary Figure 1 (c), the HDI-95 [0.0540, 0.4460] is just to the right of the ROPE [-0.05, 0.05].

Synthetic datasets were sampled as described above. The model specified in , i.e. using a non-committal uniform prior on $\beta_{1,i}$, was subsequently fit to each synthetic dataset. We computed the proportion of times out of 100 simulations (i.e. 100 synthetic datasets) the HDI-95 from the posterior over $\beta_{1,i}$ excluded the ROPE. With 23 patients, of whom 73.91% (26.09%) have three (four) longitudinal observation, the HDI-95 completely lay to the right of the ROPE 62% of the time. Keeping the sample size N to 23 but generating synthetic datasets where all subjects are sampled 5 times over the trajectory of their symptoms' improvement, the fraction of time the HDI-95 excluded the ROPE rose to 75%. Lastly, keeping the number of longitudinal samples across subjects unvaried from that observed in our cohort but increasing N to 50, the HDI-95 excluded the ROPE 71% of the times.

Supplementary figures

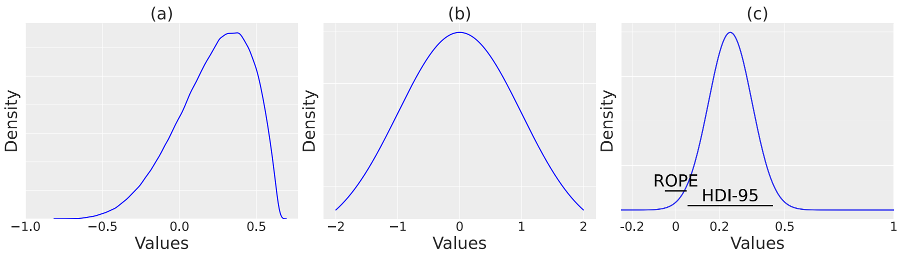

Supplementary Figure 1: **Priors for lnRMSSD rate of change with respect to symptoms' improvement** Probability density function of the priors on $\beta_{1,i}$ used in sensitivity analyses.

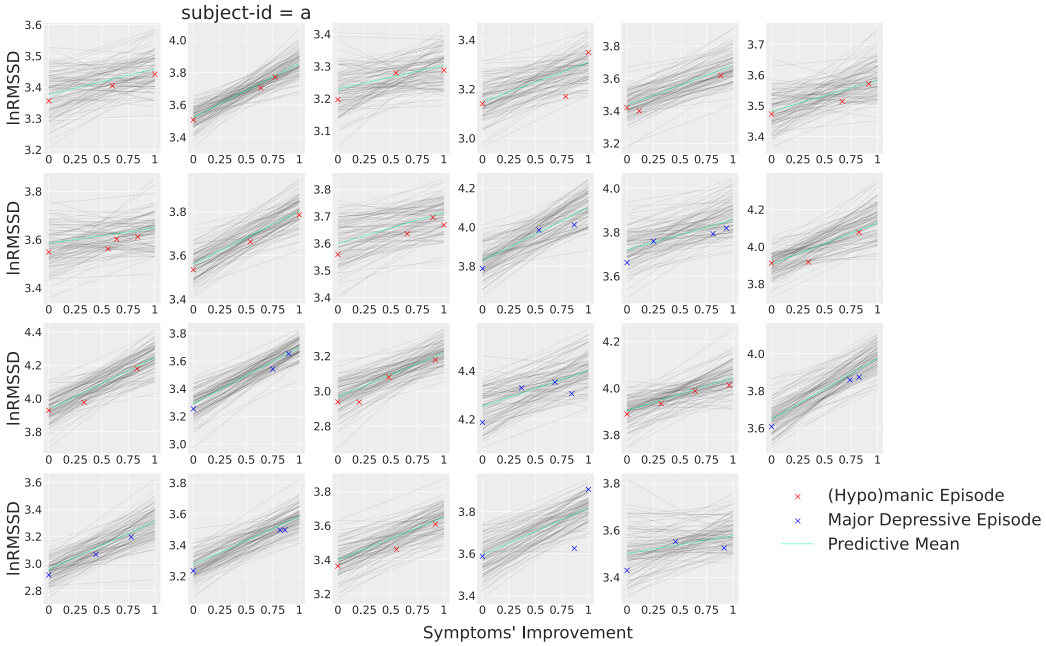

Supplementary Figure 2: **Posterior distribution from the *one-disease-model* over expected lnRMSSD values as a function of symptoms' improvement**. All subjects included in our are analyses are herewith shown. Red (blue) crosses indicates observed lnRMSSD values in patients on a mania (major depression) episode. The subplot with heading "subject-i=a" is the same as the one shown in Figure 4c. Within each subplot corresponding to a given subject in the cohort, each black line (a total of one hundred is herewith displayed to avoid clutter) represents a single draw from the posterior, while the dashed green line is the average across all black lines sampled from the posterior.

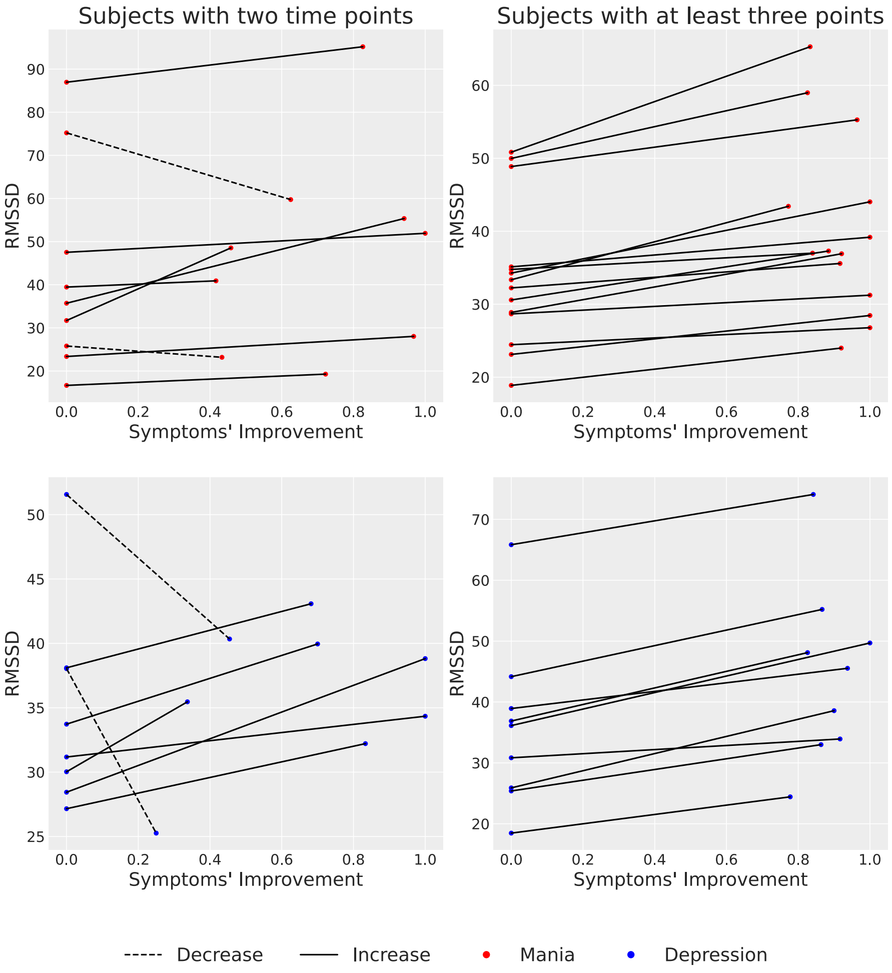

Supplementary Figure 3: **Trends in RMSSD when considering subjects from the TIMEBASE/INTREPIBD cohort with at least two measurements**. Plots on the left hand-side shows subjects with only two measurements available, while those on the right hand-side subjects with a minimum of three measurements, i.e. the very same subjects used for the main analyses and depicted in Supplementary Figure 2. For this latter group of subjects, we just retained the first and the last measurement available to avoid clutter and aid direct comparison. A positive trend in RMSSD as symptoms improve can be seen in subjects with just two RMSSD measurements available.

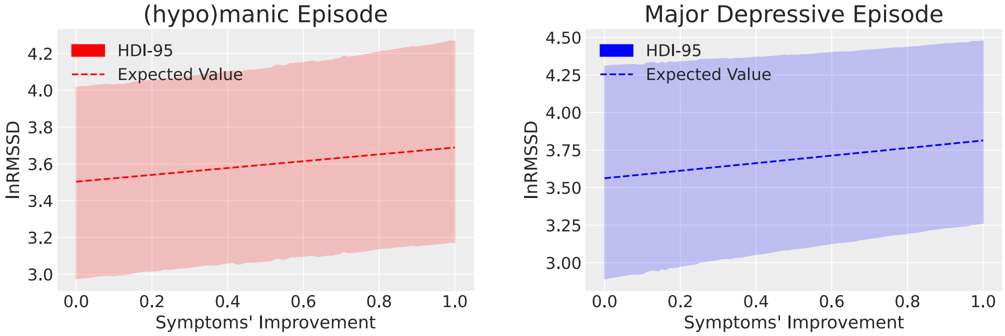

Supplementary Figure 4: **Posterior distribution from the *one-disease-model* over expected lnRMSSD values as a function of symptoms' improvement aggregated by episode's polarity**. The plot on the left (right) is obtained aggregating posterior draws from subjects recruited at the onset of a manic (major depressive) episode. The dashed line corresponds to the average of lnRMSSD expectations, i.e. the average across samples for the mean μ of the Gaussian in Equation (2), shown as a function of symptoms' improvement; the area shaded in red (blue) indicated the HDI-95 for the lnRMSSD expectation. Note that the *one-disease-model* is blind to information regarding episode polarity and was preferred, based on the WAIC, to the *two-polarities-model* which explicitly encodes the episode polarity.

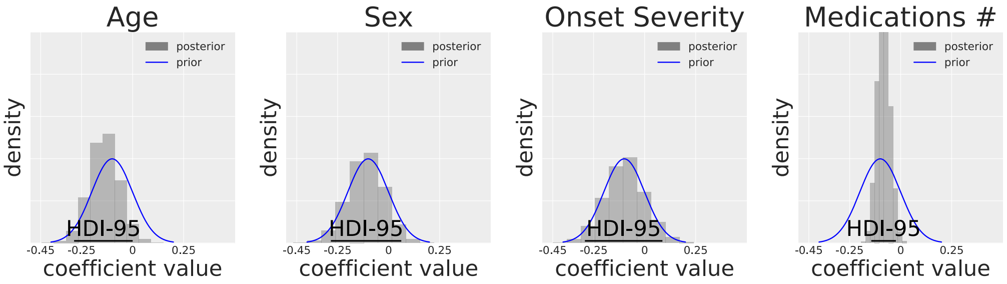

Supplementary Figure 5: **Posterior distribution from the *one-disease-model* over co-variate coefficients**. The histogram, obtained from posterior samples, is normalized so that the area under the curve sums to one. Superimposed is the density of the prior, i.e. a Gaussian with mean and standard deviation of -0.1 and 0.1 respectively. Notice that the posterior for the coefficient associated with the number of medications, sharpened around a narrower range of values relative to the posterior for other co-variates and its HDI-95 excluded the 0 value, suggesting a significant effect of medications number on lnRMSSD changes.

Supplementary tables

Supplementary Table 1: **Nighttime sleep (hours) across episode polarities and follow-up assessments.** We herewith report the mean (sd) sleep time, in hours, dected with the algorithm by Van Hees et al. 37 during the 10 pm and 5 window on the first day of the recording.

	t_0	t_1	t_2	t_3
	MEAN (STD)	MEAN (STD)	MEAN (STD)	MEAN (STD)
**MANIA**	5.65 (0.69)	5.12 (0.34)	5.45 (0.76)	5.89 (0.61)
**DEPRESSION**	5.34 (0.45)	5.59 (0.58)	5.12 (0.63)	5.71 (0.24)

Supplementary Table 2: **Medications by class across manic and depressive episodes for each longitudinal assessment.** Each cell shows the average (standard deviation) number of medications under a given drug class for patients across manic episode (ME) and major depressive episode (MDE) during each of the follow-up assessments, $t \in \{t_0, t_1, t_2, t_3\}$. Li : lithium; SSRI: selective serotonin reuptake inhibitors; SNRI: serotonin and norepinephrine reuptake inhibitors; TCA: tricyclics; MAOI: monoamine oxidase inhibitors; OAD: other antidepressants; AP1: first generation antipsychotic; AP2 second generation antipsychotic; AED: antiepileptic drug; AMP: amphetamines; AH: antihistamines; AAD: antiarrhythmic drug; AC: other anticholinergic medications; BDZ: benzodiazepines

		**LI**	**SSRI**	**SNRI**	**TCA**	**MAOI**	**OAD**	**AP1**	**AP2**
t_0	ME	0.64 (0.5)	0 (0)	0 (0)	0 (0)	0 (0)	0 (0)	0.21 (0.43)	1 (0)
	MDE	1 (0)	0.22 (0.44)	0.33 (0.5)	0.11 (0.33)	0 (0)	0.22 (0.44)	0.11 (0.33)	0.67 (0.5)
t_1	ME	0.85 (0.36)	0 (0)	0 (0)	0 (0)	0 (0)	0 (0)	0.21 (0.43)	1 (0)
	MDE	0.88 (0.33)	0.44 (0.52)	0.11 (0.33)	0 (0)	0 (0)	0.11 (0.33)	0.11 (0.33)	0.78 (0.44)
t_2	ME	0.85 (0.36)	0 (0)	0 (0)	0 (0)	0 (0)	0 (0)	0.14 (0.36)	0.86 (0.36)
	MDE	0.88 (0.33)	0.33 (0.11)	0.11 (0.33)	0 (0)	0 (0)	0.44 (0.22)	0 (0)	0.67 (0.5)
t_3	ME	1 (0)	0 (0)	0 (0)	0 (0)	0 (0)	0 (0)	0.25 (0.5)	0.5 (0.57)
	MDE	0.5 (0.71)	0 (0)	0 (0)	0 (0)	0 (0)	0.5 (0.71)	0 (0)	0.5 (0.71)

		**AED**	**β-BLOCKER**	**OPIOD**	**AMP**	**AH**	**AAD**	**AC**	**BDZ**
t_0	ME	0.36 (0.50)	0 (0)	0 (0)	0 (0)	0 (0)	0	0.21 (0.42)	0.43 (0.51)
	MDE	0.44 (0.52)	0.11 (0.33)	0 (0)	0 (0)	0 (0)	0 (0)	0.11 (0.33)	0.44 (0.52)
t_1	ME	0.36 (0.50)	0 (0)	0 (0)	0 (0)	0 (0)	0 (0)	0.07 (0.27)	0.64 (0.50)
	MDE	0.55 (0.53)	0.11 (0.33)	0 (0)	0 (0)	0 (0)	0 (0)	0 (0)	0.55 (0.52)
t_2	ME	0.21 (0.43)	0 (0)	0 (0)	0 (0)	0 (0)	0 (0)	0.07 (0.27)	0.43 (0.51)
	MDE	0. 55 (0.53)	0.11 (0.33)	0 (0)	0 (0)	0 (0)	0 (0)	0 (0)	0.44 (0.53)
t_3	ME	0 (0)	0 (0)	0 (0)	0 (0)	0 (0)	0 (0)	0 (0)	0.5 (0.57)
	MDE	0.5 (0.71)	0.5 (0.71)	0 (0)	0 (0)	0 (0)	0 (0)	0 (0)	0 (0)

Supplementary Table 3: **Clinical-demographic features of patients on an acute manic or depressive episodes excluded from analysis as not providing a minimum of three samples** "Medications #" refers to the number of drugs recorded in our cohort with a known influence on HRV which subjects were taking at the moment of study admittance. The figures herewith shown refer to the first assessment (acute episode onset), when patients were surveyed twice.

	AGE	FEMALES	MEDICATIONS #	BASELINE SYMPTOMS' SEVERITY
	MEAN (STD)	N (PERCENTAGE)	MEAN (STD)	MEAN (STD)
**MANIA** N=23	40.30 (14.87)	17 (73.91%)	3.04 (1.08)	YMRS 19.61 (8.27)
**DEPRESSION** N=21	52.38 (11.34)	15 (71.42%)	3.43 (1.56)	HDRS 18.85 (5.42)
